# The impact of vitamin E and/or selenium dietary supplementation on growth parameters and expression levels of the growth-related genes in broilers

**DOI:** 10.1186/s12917-021-02963-1

**Published:** 2021-07-21

**Authors:** Olla A. Khalifa, Rasha A. Al Wakeel, Shabaan A. Hemeda, Mohamed M. Abdel-Daim, Ghadeer M. Albadrani, Ahmad El Askary, Sabreen E. Fadl, Fatma Elgendey

**Affiliations:** 1 Department of Animal Wealth Development, Genetics and Genetic engineering, Faculty of veterinary medicine, Benha University, Benha, Egypt; 2 Department of physiology, Faculty of Veterinary Medicine, Kafrelsheikh University, Kafrelsheikh, Egypt; 3 Department of Animal Husbandry and Animal Wealth Development, Faculty of Veterinary Medicine, Alexandria University, Alexandria, Egypt; 4 Pharmacology Department, Faculty of Veterinary Medicine, Suez Canal University, Ismailia, 41522 Egypt; 5 Department of Biology, College of Science, Princess Nourah bint Abdulrahman University, Riyadh, Saudi Arabia; 6 Department of Clinical Laboratory Sciences, College of Applied Medical Sciences, Taif University, P.O.Box 11099, Taif, 21944 Saudi Arabia; 7 Biochemistry Dept., Faculty of Veterinary Medicine, Matrouh University, Matrouh, Egypt

**Keywords:** Biochemistry, Broilers, Gene expression, Growth performance, Selenium, Vitamin E

## Abstract

**Background:**

Broilers are continuously stressed because of the rapid growth rate and the environmental issues associated with industrialized poultry production systems, which lead to higher susceptibility for infection with pathogens. It is well known that vitamin E (Vit. E) and selenium (Se) supplementation have protective functions in such stressful conditions. This protocol was to investigate the impact of Vit. E and/or Se on the production performance, some serum biochemistry, and expression of some growth-related gene in the liver tissue of the broilers. The day-old chicks were allotted into four groups according to the supplement; Control group and groups supplemented with Vit. E and/or Se into Vit. E group (100 mg Vit. E/kg diet), Se group (0.3 mg sodium selenite/kg diet), and Vit E + Se group that supplemented with both Vit. E and Se.

**Results:**

The data of the present experiment showed that dietary inclusion of Vit. E and/or Se significantly (*P* ≤ 0.05) improved the production parameters without any side effect on the general health status of the broilers, which indicated by normal serum biochemical parameters. Moreover, the treatments positively affected the expression of some genes related to growth performance including growth hormone receptor (GHR) and insulin-like growth factor 1 (IGF1) in the liver tissue of broilers.

**Conclusion:**

Dietary supplementation of Vit. E and/or Se improved the production parameters and upregulate the growth-related genes without effect on the general health status of the broilers.

## Background

The production of global poultry has increased dynamically from 1 year to another [1]. This increase is attributed to good quality with low price as well as the high level of safety of the poultry meat when compared to other meat types [2]. Moreover, the short cycles of poultry production [3]. The above-mentioned reasons increasing the demand for poultry meat, which leads to an increase in the volume of production [4]. To obtain high production rate with high-quality poultry meat, it is very important to formulate a diet of high quality. The formulated diet is considered one of the key factors that affect the quantity and quality of poultry meat. Moreover, the formulated diet affecting also feed intake, feed conversion ratio, and weight gain [5].

Broilers are continuously stressed because of the rapid growth rate and the environmental issues associated with industrialized poultry production systems [6], which lead to higher susceptibility for the infection with pathogens [7]. It is well known that vitamin E supplementation has a protective function in such stressful conditions [8]. Vitamin E is a fat-soluble vitamin, known to be a biological membranous lipid component, and natural antioxidant [9]. Fundamentally vitamin E is located in the membrane lipid bilayer hydrocarbon part towards the membrane interface near to oxidase enzymes, which initiate free radicals production [10]. These free radicals are generated as a result of normal cell activity and increase due to several stress factors [11]. Thus, vitamin E protects cells and tissues from free radicals that cause oxidative damage to the cells [12]. Moreover, vitamin E is considered a natural antioxidant found in food and known as alpha-tocopherol [11] and National Research Council (NRC) [13] currently recommends 10 IU of vitamin E/kg diet to satisfy poultry’s nutritional requirements.

Selenium (Se) is an essential trace element that essential for the composition of the glutathione peroxidase enzyme, which reduces hydrogen peroxide and lipid hydroperoxides to the corresponding alcohols [14, 15]. Selenium supplementation was usually shown to be an important factor in livestock diets [16, 17]. The requirement of Se for broilers throughout the growth period is 0.15 mg/kg according to NRC [13].

Vitamin E and selenium are considered the line of defense against free radicals and till now the synergism between them is not clear. Thus, this protocol was designed to know the impact of the high level of vitamin E and/or Se supplement on the performance of broilers, some serum biochemical, and expression of the growth-related gene in the liver tissue.

## Results

### Growth performance

Table 1 show the effect of vitamin E and/or selenium dietary feed supplementation on the body weight of broilers. The table shows that dietary supplementation of vitamin E and selenium (Vit. E + Se group) significantly increased (*P* ≤ 0.05) body weight all over the experimental period when compared with the control, Vit. E, and Se groups. Moreover, the supplementation of vitamin E alone (Vit. E group) significantly increased (*P* ≥ 0.05) body weight when compared with the control and Se groups from the second week until the end of the experimental period. Meanwhile, the body weight significantly increased (*P* ≤ 0.05) in the Se group when compared with the control group. Thus, the results showed the synergistic effect of both vitamin E and selenium.

**Table 1:** Effect of dietary vitamin E and/or selenium supplementation on the body weight (g) of broilers

Groups Items	Control	Vit. E	Se	Vit. E + Se
Initial weight (g)	47.47±0.48	47.60±1.29	47.33±0.87	48.13±1.50
1 week	140.53±1.63 ^c^	152.00±1.15 ^b^	150.00±2.31^b^	160.00±2.89 ^a^
2 weeks	334.27±1.33^d^	384.00±2.00^b^	352.00±1.15^c^	420.67±2.91^a^
3 weeks	679.07±1.75^d^	737.33±0.88^b^	724.33±2.33^c^	747.1±1.76^a^
4 weeks	1095.87±2.31^d^	1196.67±2.40^b^	1185.00±2.89 ^c^	1276.67±3.33^a^
5 weeks	1682.27±1.19^d^	1850.00±2.89 ^b^	1721.33±1.86 ^c^	1890.00±2.89^a^

Values are means ± standard error. Mean values with different letters at the same row significantly at (*P*≤0.05). Vit. E= group supplemented with vitamin E; Se= group supplemented with selenium; Vit. E + Se=group supplemented with vitamin E and selenium

Table 2 show the effect of vitamin E and/or selenium dietary feed supplementation on body weight gain at different weeks. At all weeks except 3 weeks, the table shows that dietary supplementation of vitamin E and selenium (Vit E + Se group) significantly increased (*P* ≤ 0.05) body weight gain when compared with the control, Vit. E, and Se groups. Meanwhile, the body weight gain was significantly (*P* ≤ 0.05) decreased at 3 weeks in the Vit. E + Se group when compared with the control, Vit. E, and Se groups. Moreover, the supplementation of vitamin E (Vit. E group) or Se (Se group) significantly increased (*P* ≥ 0.05) body weight gain when compared with the control group. The total body weight gain significantly increased (*P* ≤ 0.05) in the Vit. E + Se group when compared with the control, Vit. E, and Se groups. Moreover, the supplementation of vitamin E alone significantly (P ≥ 0.05) increased body weight gain when compared with the control and Se groups. Meanwhile, Se supplementation alone significantly (P ≥ 0.05) increased body weight gain when compared with the control group.

**Table 2:** Effect of dietary vitamin E and/or selenium supplementation on the body weight gain (g) of broilers

Groups Items	Control	Vit. E	Se	Vit. E + Se
1 week	93.07±2.01 ^c^	104.40±1.06 ^ab^	102.67±2.36 ^b^	111.87±3.82 ^a^
2 weeks	193.73±1.55^c^	232.00±1.15^b^	202.00±1.15^c^	260.67±5.17^a^
3 weeks	344.80±2.57^c^	353.33±2.33^b^	372.33±1.20^a^	326.67±1.33^d^
4 weeks	416.80±4.01^c^	459.33±2.03^b^	460.67±5.21 ^b^	529.33±1.76 ^a^
5 weeks	586.40±3.27^c^	653.33±4.91 ^b^	536.33±4.48 ^d^	613.33±1.67^a^
Total BWG	1634.80 ±1.06^d^	1802.40 ±2.71^b^	1674.00 ±1.36^c^	1841.87± 3.91 ^a^

Values are means ± standard error. Mean values with different letters at the same row significantly at (*P*≤0.05). Vit. E= group supplemented with vitamin E; Se= group supplemented with selenium; Vit. E + Se=group supplemented with vitamin E and selenium

Table 3 show the effect of vitamin E and/or selenium dietary feed supplementation on the feed intake. At the second, third, and fifth weeks, the statistical analysis of the data showed that the feed intake was significantly (*P* ≤ 0.05) increased in the control group when compared with the other groups (Vit. E, Se, and Vit. E + Se groups). In the fourth week, the feed intake significantly decreased (*P* ≤ 0.05) in the control group when compared with the other groups that received vitamin E and/or Se. The result of the total feed intake significantly increased (*P* ≤ 0.05) in the control group when compared with the other groups. It was noticed that the smallest feed intake presented in the vitamin E group supplementation.

**Table 3:** Effect of dietary vitamin E and/or selenium supplementation on the feed intake (g) of broilers

Groups Items	Control	Vit. E	Se	Vit. E + Se
1 week	119.44±3.93 ^b^	127.09±1.21 ^ab^	132.93±2.78 ^a^	122.17±1.92 ^b^
2 weeks	372.90±1.67^a^	365.13±2.57^b^	372.73±2.88^a^	354.00±1.87^c^
3 weeks	656.18±0.93^a^	619.51±1.14^c^	649.62±0.66^b^	614.97±2.22^d^
4 weeks	772.12±0.99 ^c^	745.12±2.18 ^d^	792.72±2.26^b^	802.45±2.75^a^
5 weeks	960.28±0.21^a^	912.07±1.04 ^c^	876.20±2.25^d^	923.02±1.53^b^
Total feed intake (g)	2880.91 3.18 ^a^	2768.93 1.94 ^c^	2824.28 1.28 ^b^	2816.60 3.80 ^b^

Values are means ± standard error. Mean values with different letters at the same row significantly at (*P*≤0.05). Vit. E= group supplemented with vitamin E; Se= group supplemented with selenium; Vit. E + Se=group supplemented with vitamin E and selenium

Table 4 show the effect of vitamin E and/or selenium dietary feed supplementation on the FCR at different weeks. The supplementation of vitamin E and/or selenium significantly decreased (*P* ≤ 0.05) FCR along the experimental period except in the first, third, and fifth weeks. The FCR was insignificantly (*P* ≤ 0.05) decreased in the Vit. E and Se at first week, Vit. E + Se at third week, and Se groups at fifth week when compared with the control group. The result of the total FCR significantly increased (*P* ≤ 0.05) in the control group when compared with the other groups. Moreover, the value of the FCR was improved more in the vitamin E and vitamin E with selenium supplementation.

**Table 4:** Effect of dietary vitamin E and/or selenium supplementation on the FCR of broilers

Groups Items	Control	Vit. E	Se	Vit. E + Se
1 week	1.29±0.06 ^a^	1.22±0.01 ^a^	1.29±0.02 ^a^	1.09±0.02 ^b^
2 weeks	1.93±0.02^a^	1.58±0.01^c^	1.85±0.00^b^	1.36±0.02^d^
3 weeks	1.90±0.01^a^	1.75±0.01^b^	1.75±0.01^b^	1.88±0.01^a^
4 weeks	1.85±0.02^a^	1.62±0.01^c^	1.72±0.01 ^b^	1.52±0.00 ^d^
5 weeks	1.64±0.01^a^	1.39±0.01 ^c^	1.64±0.01 ^a^	1.50±0.00^b^
Total FCR	1.76 0.00 ^a^	1.54 0.03 ^c^	1.69 0.01 ^b^	1.53 0.03 ^c^

Values are means ± standard error. Mean values with different letters at the same row significantly at (*P*≤0.05). Vit. E= group supplemented with vitamin E; Se= group supplemented with selenium; Vit. E + Se=group supplemented with vitamin E and selenium

### Serum parameters

Table 5 showed lipid profile, liver enzymes, and kidney function test in the serum of broilers supplemented with vitamin E and/or selenium. Statistical analysis of the lipid profile data appeared that vitamin E supplementation (Vit. E) and/or selenium (Se and Vit. E + Se) significantly (*p* ≤ 0.05) increase total cholesterol and HDL when compared with the control group. Moreover, HDL significantly (*p* ≤ 0.05) increased in the group that received both supplements (Vit. E + Se). On the other side, serum triglyceride and LDL not affected. Statistical analysis of the liver enzymes (ALT and AST) and kidney function (creatinine and urea) data indicated that the inclusion of vitamin E and selenium (Vit. E + Se) significantly (*p* ≤ 0.05) decreased ALT when compared with the control group. Meanwhile, AST, creatinine, and urea not affected by the supplements.

**Table 5:** Effect of dietary vitamin E and/or selenium supplementation on the serum parameters of broilers

Groups Items	Control	Vit. E	Se	Vit. E + Se
Cholesterol (mg/dl)	140.47±3.55 ^b^	183.47±3.54 ^a^	171.60±4.65 ^a^	182.80±3.80 ^a^
Triglycerides (mg/dl)	47.56±0.29	48.71±2.14	47.98±1.65	46.35±1.52
LDL (mg/dl)	13.11±0.72	12.90±3.07	12.52±1.41	12.15±1.16
HDL (mg/dl)	97.29±1.85^c^	117.93±1.59^b^	112.67±2.03^b^	142.40±1.55^a^
Urea (mg/dl)	4.20±0.05^ab^	3.53±0.08^b^	4.93±0.45 ^a^	4.46±0.25 ^a^
Creatinine (mg/dl)	0.40 ±0.09	0.54 ±0.06	0.56± 0.05	0.46 ±0.02
ALT (u/l)	7.32±0.74 ^a^	5.96±0.52^ab^	6.13±0.03 ^ab^	4.93±0.31^b^
AST (u/l)	195.57±3.96	203.67±2.34	193.80 ±3.58	203.13 ±3.93

Values are means ± standard error. Mean values with different letters at the same row significantly at (*P*≤0.05). Vit. E= group supplemented with vitamin E; Se= group supplemented with selenium; Vit. E + Se=group supplemented with vitamin E and selenium

### Expression levels of liver growth-related gene

**Fig. 1:**
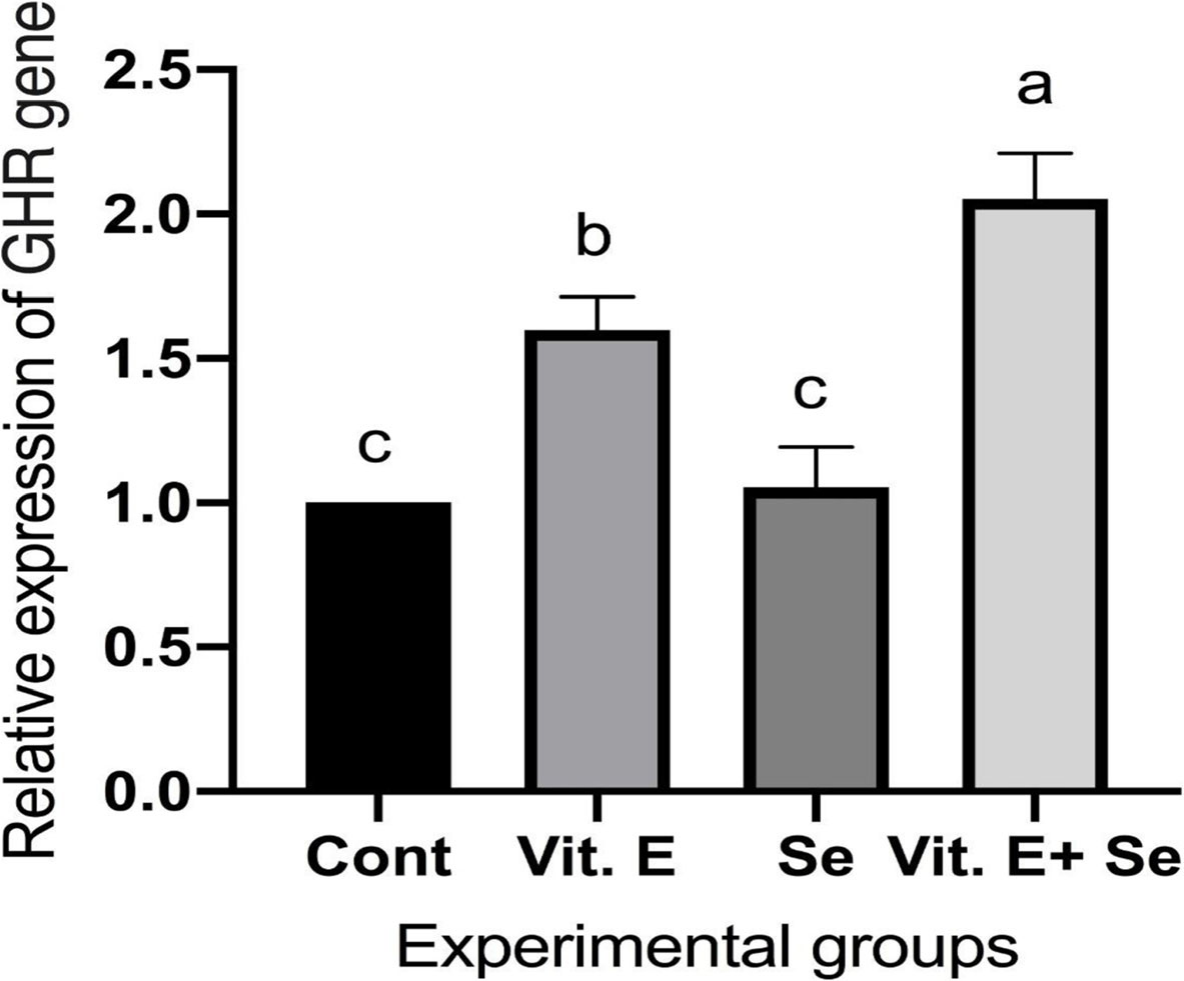
Effect of dietary supplementation with vitamin E and/or selenium on liver GHR mRNA transcript level of broilers feed on diet containing Vit E with or without Se. Values were expressed as Mean ± SE. Columns with different litters indicates statistically significant values with *P*-values < 0.01

**Fig. 2:**
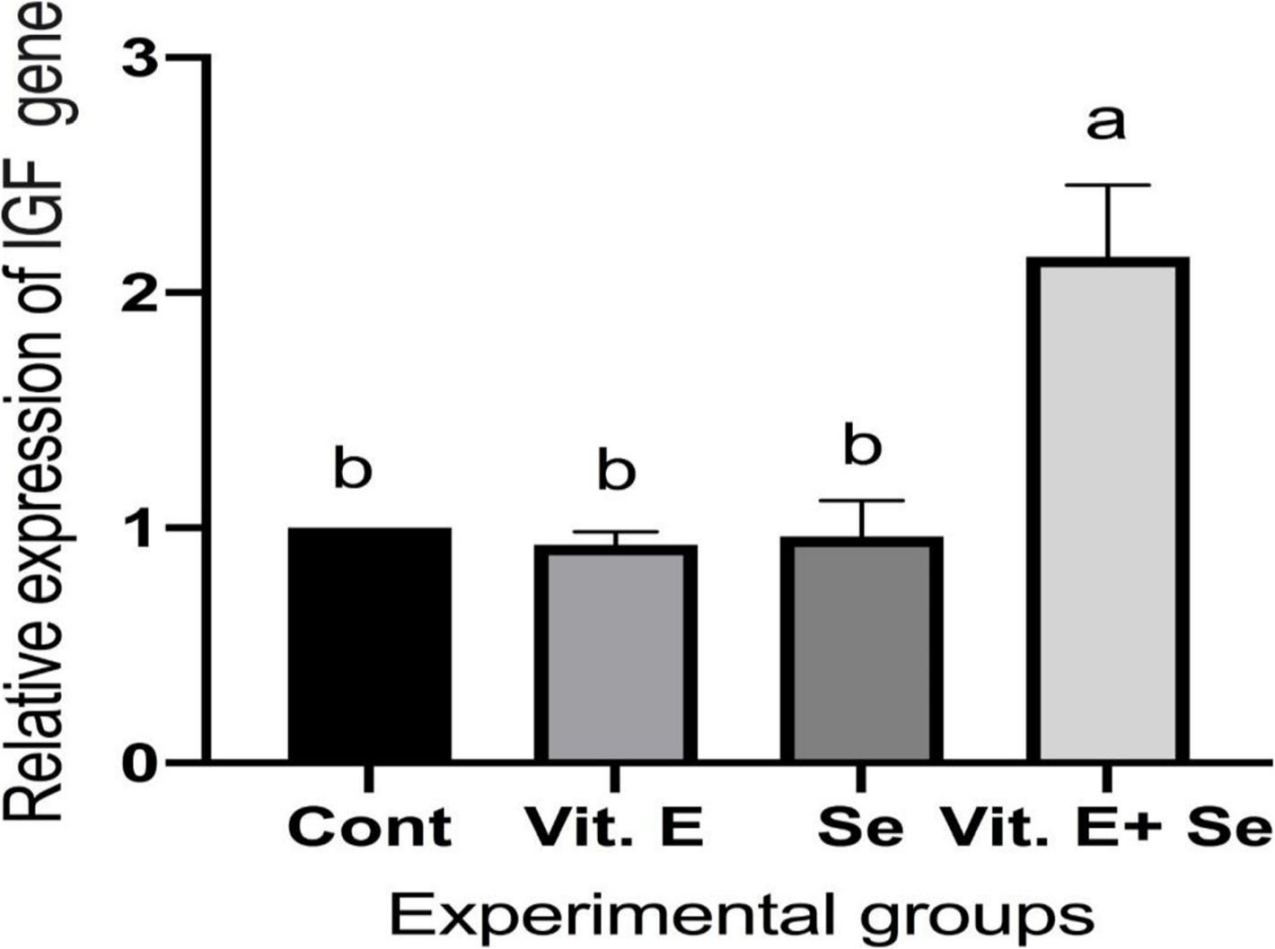
Effect of dietary supplementation with vitamin E and/or selenium on liver IGF1 mRNA transcript level of broilers feed on diet containing Vit E with or without Se. Values were expressed as Mean ± SE. Columns with different litters indicates statistically significant values with *P*-values < 0.01

Regarding results of the growth-related gene, the addition of vitamin E and selenium induced change in the expression pattern of growth hormone receptor (GHR) and insulin-like growth factor 1 (IGF1). Where the group fed by diet with vitamin E and selenium (Vit. E + Se) showed a significant increase in the levels of these growth-related genes compared to the other groups, which is revealed by the upregulation of GHR (Fig. 1) and IGF1 (Fig. 2) genes. On the other side, the group fed vitamin E without selenium (Vit. E) recorded a significant increase in the level of the GHR gene compared to the control and selenium (Se) groups, which is revealed by the upregulation of the GHR gene (Fig. 1).

## Discussion

Excessive dietary Vitamin E and selenium have been researched in order to improve the growth of the broilers and investigate the effect of Vit. E and/or Se on gene expression of the growth related gene in the liver tissue. The combination of both supplements (Vit. E and Se) has shown an increase in the body weight and weight gain of broilers [18, 19]. These results are in line with Salahuddin et al. [20] who showed that dietary vitamin E and selenium (100 mg and 0.22 mg/kg diet for vitamin E and selenium, respectively) improves the live body weight and gain. These findings may be due to the enhancement of dietary nutrient digestion and assimilation by 100 mg vitamin E and 0.3 mg of Selenium per kg diet. Furthermore, the antioxidant properties of both vitamin E and selenium, which affect the health of the birds and so increased body weight. These results are dissonant with Habibian et al. [21] who found that supplementing vitamin E and selenium has no impact on the broiler’s growth, except feed intake, which decreases with treatments. Moreover, dietary vitamin E or selenium improved the weight of broilers compared with those who didn’t receive vitamin E or selenium (control group). These results may be attributed to origin of the selenium used in the study (sodium selenite) where the bio-efficacy of selenium depends on its chemical form. Surai et al. [22] reported that organic selenium is better than the traditional inorganic form of the selenium. Also,

Swain et al. [23] reported that broilers supplemented with vitamin E consumed less feed than those fed the basal diet. These results come in constant with the results of the feed intake. Also, Pompeu et al. [24] reported there is no link between supplementing dietary vitamin E and growth performance. Yoon et al. [25] found that the addition of selenium (*P* > 0.05) did not impact the growth of broilers at 42 d of age. Dietary selenium or vitamin E supplements decrease mortality and increase body weight gain of non-vaccinated broilers experimentally infected with *E. tenella* [26].

On the other side, the results of the total feed intake in this experiment are in line with Dalia et al. [27] who found that vitamin E and/or Se (inorganic or organic) inclusion significantly decreases feed intake and improves growth parameters. These results may be attributed to the good health of the broilers as a result of the antioxidant effect of vitamin E and/or Se. In contrast, Salahuddin et al. [20] reported that the broiler feed intake was not affected by additional vitamin E and selenium. Vitamin E and Se supplements had no effect on feed use and appeared to promote growth [28]. Likewise, Arrieta [29] revealed that there is no significant between different groups treated with vitamin E and/or Se related to feeding intake. Regarding results of FCR, Choct et al. [30] reported a significant decrease in the feed intake while the same weight gains are preserved or increased this leads to a decrease in FCR. Thus, the FCR results were significantly improved as a result of a significant decrease in feed intake with increased weight gain. Similar results were obtained by Ziaei and Pour [31]. These results are discordant with Habibian et al. [21] who found FCR of broilers, which received different vitamin E and selenium levels didn’t change during the entire experimental period. Moreover, Tayeb and Qader [32] said there are no major variances among all treatments and the controls at 49 days of age in live body weight, body weight gain, feed intake, and feed conversion ratio of broilers treated with Vit. E and/or Se. On the other side, Choct et al. [30] found that any increase in selenium levels in the broilers diet would decrease FCR. In addition, Selvam et al. [33] indicated the addition of vitamin E to broilers subjected to high stocking density (HSD) at 70 g/ton of feed could effectively reverse the negative consequences of HSD and increase broiler production. The results of the products were confirmed by the results of the gene expression of the growth-related genes.

These results of the production performance were confirmed by the results of the serum biochemistry, which done indication about the health status of the birds. This improvement in serum biochemistry may be attributed to the antioxidant effects of vitamin E and/or selenium. Habibian et al. [15] found the same result as indicated in the present investigation and reported that LDL and HDL decrease and increase, respectively, in broilers fed on a diet containing vitamin E. Vitamin E supplementation decrease the activity of AST and ALT [34]. In addition, the combination of vitamin E and Se can mitigate the side effects of afla and Ochratoxin on the serum biochemistry of broilers [35]. On the other hand, Arslan et al. [36] reported that there are no important variations between control and experimental groups in plasma cholesterol, triglyceride, AST, or ALT. Moreover, Hala and Fathy [37] found a significant decrease in cholesterol, triglyceride as well as LDL levels with increase HDL in the serum of broilers supplemented with different levels of Se. In our results the cholesterol increased with the vitamin E and/or selenium supplementation, which may be attributed to the level of vitamin E and/or selenium used in the present study. These results confirmed by the result of Habibian et al. [15]. The cause of this result not clear. However, this obviously requires more study.

To our knowledge, there is no literature about the effect of vitamin E and/or Se on the expression of the growth-related gene in broilers. There are many papers that explain the relationship between vitamin E and/or Se and the expression of the antioxidant-related gene. The results of the expression of the growth-related gene in the present design showed upregulation of the growth hormone receptor (GHR) and insulin-like growth factor 1 (IGF1). These results may be attributed to the antioxidant effect of vitamin E and/or Se, which confirmed through many papers [38–40]. The broilers GHR play an important role in broilers’ production parameters because of its cardinal role in the development [41]. Moreover, the full growth and development of birds are mainly connected to the pathways of “the hypothalamus-hypophysical pathway” [42]. Both hormones; Growth hormone and somatostatin are secreted from neurohypophysis, which plays a double role in the modulation and control of the pituitary and growth hormone (GH). Growth hormone circulates back into the liver through the blood and binds on the liver cell surface with the GH receptor (GHR) in order to initiate signaling mechanisms that encourage the expression of the IGFs. IGFs circulate through the blood into the body tissues and stimulate growth and differentiation of the cell [43].

## Conclusion

Our data investigated that the dietary inclusion of Vit. E and/or Se significantly (*P* ≤ 0.05) improved the production parameters without any side effect on the general health status of the broilers, which indicated by normal serum biochemical parameters. Moreover, the treatments positively affected the expression of some genes related to growth performance including growth hormone receptor (GHR) and insulin-like growth factor 1 (IGF1) in the liver tissue of broilers.

## Methods

### Experimental protocol

**Fig. 3:**
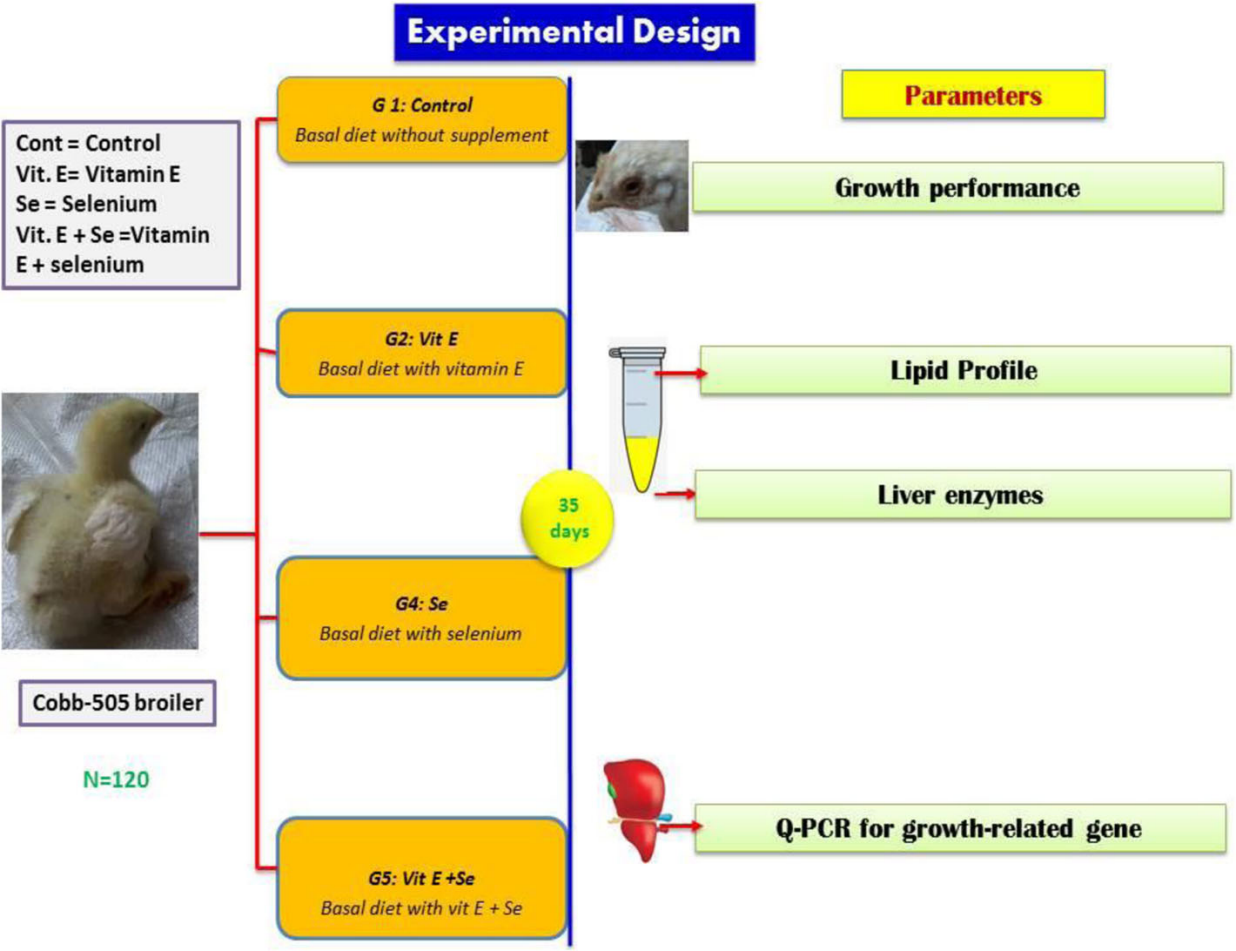
Showed experimental design of the experiment

To investigate the effect of vitamin E (alpha-tocopherol acetate from Sigma Al-drich Co., USA) and/or sodium selenite (from Eibico Company, Egypt) on broilers’ growth performance for 35 days, 120 one day-old chicks (Cobb-505) with average weight 47.63 ± 0.17 g/chick were used. The obtained chicks from a local farm were reared in a clean, well-ventilated room and received feed and water ad libitum with good sanitation and hygienic management. The birds were reared on sawdust bedding on the floor in replicate where the replicates were separated by wire (surrounded by wood and its height about 2 m) with light/dark 23/1 h. /day. The day-old chicks were allotted randomly (ranking method) into four groups (Fig. 3) according to the supplement; Group without supplement (control group) that fed on a prepared (Table 6) basal diet (according to broiler nutrition specification, 2007) and three groups supplemented with vitamin E and/or selenium; Vit. E group that supplemented with vitamin E alone (100 mg Vit. E/kg diet), Se group that supplemented with selenium alone (0.3 mg sodium selenite/kg diet), and Vit. E + Se group that supplemented with both vitamin E and selenium (100 mg Vit. E + 0.3 mg sodium selenite/kg diet). Each group had three replicates (ten chicks/replicate). The supplements were added to the formulated diet at different stages of broilers growth (starter, grower, and finisher). The birds were vaccinated against ND and IBD at 7 & 12 day, respectively according to the program of vaccination during the experimental period.

**Table 6:** Physical and chemical constituent of the basal diet

	Starter	Grower	Finisher
**Ingredients %**			
Corn	58.56	63.53	67.9
SBM, 47.5	30	29.1	26
CGM	6	2	1
Soya oil	1.5	2	2
Di-calcium phosphate	1.63	1.45	1.38
Limestone	1.12	0.86	0.77
Salt	0.43	0.38	0.375
Lysine HCL	0.29	0.19	0.13
Dl Methionine	0.17	0.19	0.145
Premix^a^	0.3	0.3	0.3
**Nutrients %**			
ME (kcal/kg)	3096	3124	3155
Crude protein	23	20.35	18.55
Lysine	1.325	1.19	1.05
Methionine	0.597	0.54	0.47
Methionine+Cyst	0.982	0.89	0.78
Meth+Cys/lysine ratio	0.74	0.74	0.74
Crude fat	4.15	4.74	4.86
Ca	0.9	0.86	0.8
AP	0.45	0.42	0.4

^a^Premix provides Vit A (12,000 Iu), vit D (5000 Iu), vit E (50 mg), vit K3 (3 mg), vit B1 (3 mg), vit B2 (8 mg), vit B6 (4 mg), vit B12 (0.016 mg), nicotinic acid (60 mg), pantothinic acid (15 mg), folic acid (2 mg), biotin (0.2 mg), iron (40 mg), copper (16 mg), zinc (100 mg), manganese (120 mg), iodine (1.25 mg), selenium (0.3 mg) per 1 kg diet

The weight of the individual broiler was taken at the first of the protocol and weekly for the calculation of the growth parameters according to Vohra and Roudybush [44], Castell and Tiews [45], and Tacon [46] for (body weight (BW), weight gain (WG), and feed conversion ratio (FCR), respectively. Meanwhile, the feed intake/replicate was recorded weekly.

Growth parameters: Body weight, weight gain, feed conversion ratio (FCR) were calculated.

$$ \mathrm{Weight}\ \mathrm{gain}=\left(\mathrm{Final}\ \mathrm{body}\ \mathrm{weight}-\mathrm{Initial}\ \mathrm{body}\ \mathrm{weight}\right) $$

Total weight gain (TG) was calculated by the following equation,

$$ \mathrm{TG}\ \left(\mathrm{g}\right)=\mathrm{Wt}1-\mathrm{Wt}0 $$

Where wt1 is the final body weight (g) and wt0 is the initial body weight (g) according to Castell and Tiews [45].

Feed conversion ratio was calculated by the following equation according to Tacon [46].

$$ \mathrm{FCR}=\mathrm{Feed}\ \mathrm{intake}\ \left(\mathrm{g}\right)/\mathrm{weight}\ \mathrm{gain}\ \left(\mathrm{g}\right) $$

### Serum and tissue samples collection

After 35 days, two birds from each replicate (6 birds/ group) were randomly selected and anesthetized by intraperitoneal injection (IP) of sodium pentobarbital with a dose of 50 mg/kg for the blood (from wing vein) and liver tissue samples collection. The selection of the broilers for blood samples was not based on any pre-specified effect. The blood samples without anticoagulant (6 ml/sample) were used for serum separation at 3000 rpm for 15 min and kept at − 20 °C. The separated serum samples were used to measure liver enzymes (ALT and AST), kidney function (urea and creatinine), and lipid profile, which include total cholesterol, triglyceride, low-density lipoprotein, and high-density lipoprotein by using commercial kits from the Bio-Diagnostic Company, Giza, Egypt. The investigators were not blinded during data collection and computational analysis. Blinding was used during analysis.

At the end of the protocol, the remaining live broilers were euthanized in strong bags by CO2 suffocation. Moreover, after the collection of the tissue samples for gene expression, the remnant of the sacrificed birds and dead ones as well as bedding material was buried in the strict hygienically controlled properly constructed burial pit.

### RNA isolation and cDNA synthesis

For measuring the gene expression of growth regulatory genes, growth hormone receptors (GHR) and insulin-like growth factor (IGF), liver tissue samples were collected from five birds in each treatment and directly frozen in liquid nitrogen and stored at − 80 °C until analysis. The total RNA was extracted using TRI reagent (easy-RED™, iNtRON Biotechnology, South Korea), following the manufacturers’ protocol. The integrity and quality of RNA were verified on 2% agarose gel electrophoresis. The cDNA was synthesized from the RNA using the SensiFAST™ cDNA synthesis kit (Bioline, United Kingdom), then stored at − 20 °C until analysis (Table 7).

**Table 7:** Primers used for qRT-PCR analysis

Gene	Primer	Reference	Annealing temperature
*B-actin*	F: ACCTGAGCGCAAGTACTCTGTCT R: CATCGTACTCCTGCTTGCTGAT	Xie et al. [47]	60 °C
*GHR*	F: AACACAGATACCCAACAGCC R: AGAAGTCAGTGTTTGTCAGGG	El-Naggar et al. [48]	60 °C
*IGF*	F: CACCTAAATCTGCACGCT R: CTTGTGGATGGCATGATCT	El-Naggar et al. [48]	60 °C

*GHR* growth hormone receptor and *IGF* Insulin like growth factor

### Q-PCR (quantitative real-time polymerase chain reaction)

Real-time amplification was done in Stratagene MX300P Realtime PCR machine (Agilent Technologies, USA), using the SensiFast™ SYBR Lo-Rox kit (Bioline, United Kingdom) following the manufacturer’s recommendations. The thermocycler protocol was: initial denaturation at 95 °C for 15 min, followed by 40 cycles at 95 °C for 15 s, annealing 60 °C for 1 min. Dissociation curves were analyzed to validate those specific PCR products were amplified. All samples were tested in duplicates. Ct values of the target genes were normalized against Ct values of the house-keeping gene (β actin gene) of the sample gene, then used in the calculation of fold change(2^−ΔΔCT) according to Livak and Schmittgen [49].

### Statistical analysis

The statistical analysis of data was performed using SPSS version 20. One-way ANOVA was used to test the effect of supplementing vitamin E and/or selenium into the birds’ diet.

## Data Availability

The datasets used and/or analyzed during the current study are available from corresponding author on reasonable request.

## Abbreviations

ALT: Alanine transaminase
AST: Aspartate aminotransferase
BW: Body weight
DNA: Deoxyribonucleic acid
FCR: Feed conversion ratio
GH: Growth hormone
GHR: Growth hormone receptor
HDL: High-density lipoprotein
HSD: High stocking density
IGF1: Insulin-like growth factor 1
IP: Intraperitoneal injection
Q-PCR: Real-Time Polymerase Chain Reaction
LDL: Low-density lipoprotein
ND: Newcastle disease
NDV: Newcastle disease virus
NF: Nuclear factor
RNA: Ribonucleic acid
Se: Selenium
